# Photosynthetic base of reduced grain yield by shading stress during the early reproductive stage of two wheat cultivars

**DOI:** 10.1038/s41598-020-71268-4

**Published:** 2020-09-01

**Authors:** Hong Yang, Baodi Dong, Yakai Wang, Yunzhou Qiao, Changhai Shi, Lele Jin, Mengyu Liu

**Affiliations:** 1 Key Laboratory of Agricultural Water Resources, Hebei Laboratory of Agricultural Water-Saving, Center for Agricultural Resources Research, Institute of Genetics and Developmental Biology, Chinese Academy of Sciences, 286 Huaizhong Road, Shijiazhuang, 050021 China; 2 University of Chinese Academy of Sciences, Beijing, 100049 China; 3 College of Agronomy, Qingdao Agricultural University, Qingdao, 266109 China

**Keywords:** Agroecology, Environmental impact

## Abstract

The young microspore (YM) stage is the most sensitive period for wheat grain formation to abiotic stress. Shading stress during YM stage reduces grain yield mainly due to grain number decrease. However, the photosynthetic base for grain number decrease is still unclear. In this study, 100% (control), 40% (S1), and 10% (S2) of natural light were applied for 1, 3, 5, and 7 days (D1, D3, D5 and D7) during YM stage of two wheat cultivars (Henong825, Kenong9204). The results showed that grain number in Henong825 and Kenong9204 was reduced by − 3.6 to 33.3% and 14.2–72.7%, respectively. The leaf photosynthetic rate (Pn) in Henong825 and Kenong9204 was deducted by 4.5–93.9% and 26.4–99.0%. Under S1–D1, the leaf Pn of Henong825 reducing was mainly due to the reduction of light intensity. With shading intensity and duration increasing, the reasons for leaf Pn decrease were the low light intensity, the low Gs (stomatal conductance) and chlorophyll content, the damage of ultrastructure of chloroplast and photosynthetic system. Under S2–D7, the chlorophyll content, Fv/Fm (maximal photochemical efficiency of photosystem II) and Jmax (maximum electron transport) were reduced by 19.6%, 5.2% and 28.8% in Henong825, and by 29.9%, 7.8% and 33.1% in Kenong9204. After shading removal, the leaf Pn of Kenong9204 under D5 and D7 could not reach to the level of CK. This study indicated that the reduction of leaf Pn was mainly due to the low light intensity under short shading duration (shorter than 3 days), and due to low light intensity and damage of the leaf photosynthetic system under longer shading duration (longer than 5 days), especially for Kenong9204 (shade-sensitive cultivar).

## Introduction

Light is necessary for plant growth. Solar radiation gradually decreased with climate change. According to the Intergovernmental Panel on Climate Change (IPCC)^1^, the global radiation reaching the earth’s surface from 1960 to 2000 has been reduced by 1.3% on average per decade. In most parts of China, the solar radiation has declined by more than 6% per decade^2^, especially in South China and the North China Plain^3^. The solar radiation of this region from 1961 to 2003 annually decreased by 19.6 MJ m^−2^ a^−1^^4,5^. Moreover, the reduction of solar radiation during wheat-growing season ranged from 11 to 21% in 2010–2012^6^. Thus, in order to ensure food security in the shading conditions in the future, it is necessary to understand and assess how solar radiation affects yield variation in wheat.

Grain yield reduction due to shading depends on shading intensity and duration, growth stage and variety characteristics of plant^7,8^. From jointing (Z31)^9^ to maturity (Z90), moderate shading intensity (≥ 85% of full radiation) augmented the grain yield of shade-tolerant cultivars, but more than 22% shading intensity (≤ 78% of full radiation) significantly reduced the yield^10,11^. Shading at approximately 20 days pre-anthesis of wheat, grain yield decreased the most primarily because of the decrease in kernel numbers^12^. Fischer^13^ also found that at 15 days pre-anthesis, grain number was the greatest response of the crops to shading stress. The young microspore (YM), from the tetrad to the early microspore phase^14^, occurs 10–12 days before heading^15^. The YM stage is the phase most vulnerable to the impacts of abiotic stress on yield formation^16^. Shading stress at the YM stage reduced grain number from 40 to 90%, causing a large reduction in grain yield of 94.3%^17^. Therefore, understanding the effects of different shading intensities and durations in the YM stage on grain number is of particular interest.

Leaf photosynthesis predominantly contributes to carbohydrate accumulation and determines crop yield^18^. In the shading conditions, photosynthetic rate (Pn) significantly decreased^10^. Previous study showed that, shading during the developmental stage substantially decreased leaf Pn and hindered carbon assimilation, resulting in the reduction of yield^19,20^. However, in these studies, the reduction of Pn under shading conditions occurred in different mechanisms. The researcher believed that the limitation of Pn under shading conditions could be due to the attenuation of irradiance^21^. Other researcher indicated that the change of Gs directly affects photosynthesis^22,23^. Shading stress decreased Gs, limiting the diffusion of carbon dioxide (CO_2_) from the atmosphere into the leaves and reducing the Pn^24^. Moreover, the decline in the ratio of chlorophyll *a* and chlorophyll *b* in light harvesting, under shading conditions may also modify the Pn^25^. However, previous studies have shown that these effects of shading stress are cultivar dependent^8,26^. Chlorophyll fluorescence parameters in leaves are utilized as a baseline in investigating photosynthetic systems and reactions and are affected by shading^27^. Shading may increase the efficiency of light energy utilization and the activity of photosystem II (PSII), but it may also reduce the energy transport from PSII to photosystem I (PSI)^28^. This higher energy production by PSII in plants subjected to shading has been shown to be consumed by non-photochemical reactions, thereby reducing their photosynthetic capacity^11^. Furthermore, shading significantly reduces leaf thickness^29^ and alters the leaf tissue morphology, chloroplast morphology, and ultrastructure of plants^30–32^, so it can reduce their Pn. Nonetheless, the mechanisms underlying the photosynthetic responses of various cultivars to different shading intensities and durations are yet to be further investigated and fully elucidated.

Based on our previous experiments^17^, relatively heavy shading, in which 98% of natural light was blocked in the YM stage, critically reduced the grain number and resulting in reduction of grain yield of wheat. There were few studies on photosynthetic mechanism of reduction of grain number in YM stage. The aims of this study were to investigate the photosynthetic mechanism of grain number drop of different cultivars due to YM stage shading. It was hypothesized that, I) the grain number reduction caused by shading stress is related to the decrement in photosynthetic capacity alone or in combination with damage of photosystems. II) shade-tolerant cultivar suffers less and recovers faster (more) than shade-sensitive cultivar.

## Results

### The microclimate under shading conditions

**Figure 1:**
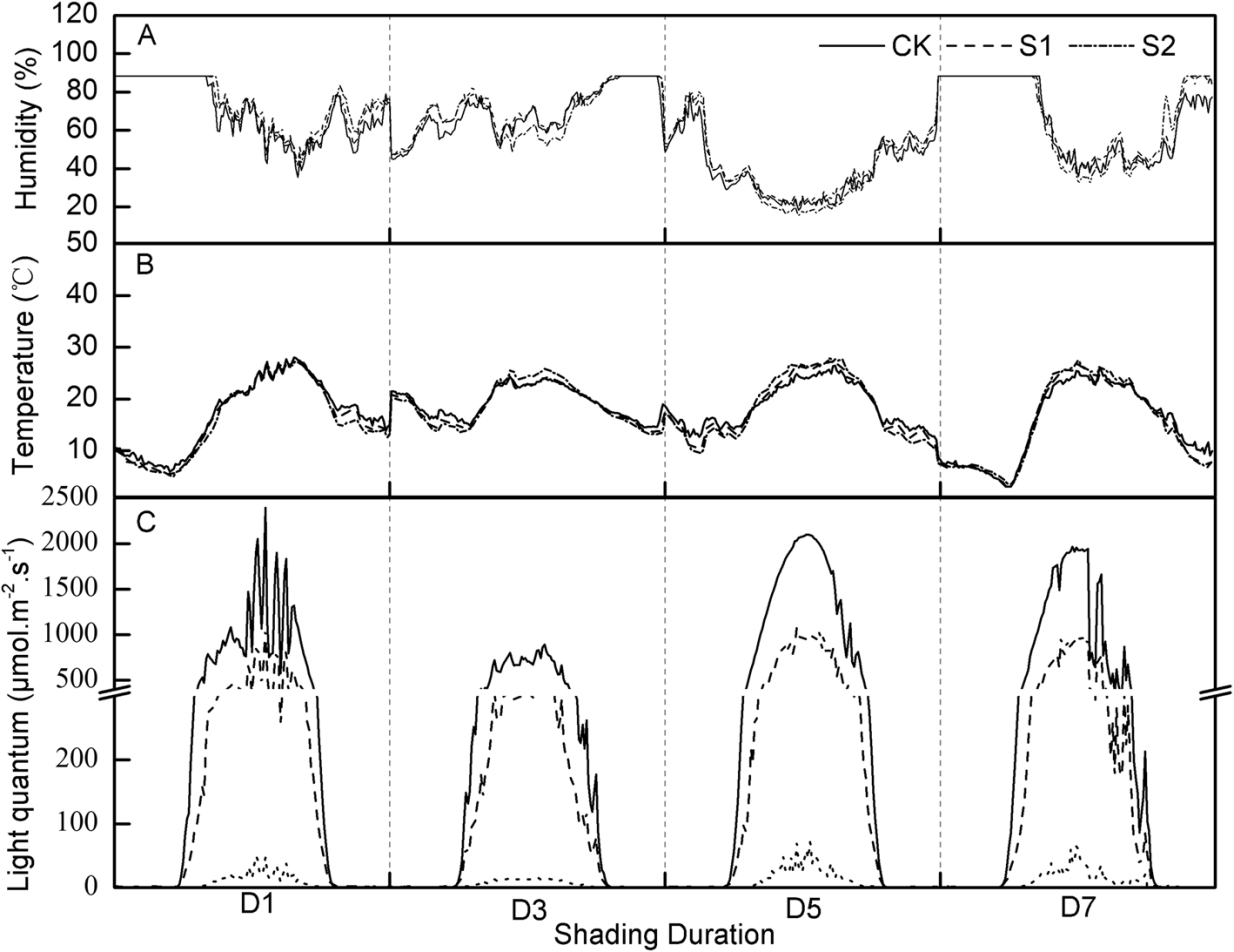
The effects of shading on the microclimate during the experimental period in 2017. CK, control with 100% of natural light; S1, 40% of natural light; S2, 10% of natural light; D1, 1 day; D3, 3 days; D5, 5 days; D7, 7 days.

There were no differences in air temperature and relative humidity among the shading treatments (Fig. 1A,B). The daytime air temperature above the wheat canopy was on average 0.4 °C higher in S1 and 0.9 °C higher in S2 than in the control. The relative humidity was on average 2.5% higher in S1 and 2.4% lower in S2 than in the control. Compared to that in full light conditions, the light intensity in S1 and S2 significantly decreased by approximately 60% and 90%, respectively (Fig. 1C). The spectral irradiance above the wheat canopy significantly decreased with the shading conditions (Table 1). Moreover, the fraction of the visible light significantly decreased in S2 but not in S1. However, there had no significant changes in the red/far red with shading conditions.

**Table 1:** The light quality under different shading treatments in 2018.

Cultivar	Treatments	T (μW cm^−2^ sr^−1^ nm^−1^)	B/T (%)	G/T (%)	R/T (%)	FR-T (%)	R/FR
Henong825	CK	18,543.7a	13.8a	15.9a	14.6a	12.4a	1.19a
	S1	7,629.9b	13.4a	15.4a	14.3a	12.1a	1.17a
	S2	193.0c	12.1b	14.3b	13.2b	11.5b	1.15a
Kenong9204	CK	17,391.2a	14.2a	16.0a	14.6a	12.2a	1.19a
	S1	9,723.6b	13.2a	15.4a	14.3a	12.2a	1.17a
	S2	164.4c	11.9b	13.4b	12.6b	11.1b	1.14a

The data were recorded during shading stress as means of three replicates. The value in each column and each cultivar followed by different letters indicate differences at *P* < 0.05. B, blue light (400–500 nm); G, green light (500–600 nm); R, red light (600–700 nm); FR, far-red light (700–800 nm); T, the total spectral irradiance (350–2,500 nm, μW cm^−2^ sr^−1^ nm^−1^); CK, control with 100% of natural light; S1, 40% of natural light; S2, 10% of natural light.

### Effects of shading on grain yield components

The shading during the YM stage had a significant effect on grain number and grain weight, resulting in decreased grain yield (Table 2). The detrimental effect on grain number and grain weight increased drastically with increasing in shading intensity and shading duration. However, the responding extent of the two cultivars was very different. The grain number of Henong825 was decreased by − 3.6% and − 1.5% in D1, by 1.7% and 13.5% in D3, by 10.7% and 14.1% in D5, and by 16.0% and 33.3% in D7, under S1 and S2 respectively. Whereas grain number of Kenong9204 was reduced by 14.2% and 22.5% in D1, by 28.3% and 48.9% in D3, by 38.3% and 63.6% in D5, and by 47.4% and 72.7% in D7. Grain weight of both cultivars was not significantly impacted by S1 no matter how long the shading lasted. It was only significantly decreased by S2 lasted more than 3 (Kenong9204) and 5 days (Henong825). And, grain weight of Henong825 was only significantly reduced by 3.5–6.6% in D5-D7. However, grain weight of Kenong9204 was significantly reduced by 2.9–26.8% in D3–D7. The reduction of grain number and grain weight jointly resulted in grain yield reduction by − 3.1 to 15.2% and − 0.9 to 37.7% in Henong825, by 14.0–47.2% and 21.4–79.4% in Kenong9204 in S1 and S2 respectively. In addition, the change in the aboveground biomass among the shading treatments was similar to that in the grain yield of both cultivars (Table 2). The harvest index (HI) of Kenong9204 significantly decreased in S1 and S2, but not in S1–D1. The HI of Henong825 did not significantly change in S1 and S2, but it significantly decreased in S2–D5 and S2–D7.

**Table 2:** The effects of shading intensities on the yield, yield components, biomass, and HI of wheat in different shading durations in 2016–2017 and 2017–2018.

Cultivar	Shading treatment		2016–2017					2017–2018				
	Duration	Intensity	Grain yield (g m^−2^)	Grain number (10^−3^ m^−2^)	Grain weight (mg grain^−1^)	Biomass (g m^−2^)	HI (%)	Grain yield (g m^−2^)	Grain number (10^−3^ m^−2^)	Grain weight (mg grain^−1^)	Biomass (g m^−2^)	HI (%)
Henong825	D1	CK	1,039.1a	25.1a	41.4a	2,230.4a	46.6a	951.4a	27.9a	34.1a	1994.4a	47.7a
		S1	1,082.1a	26.2a	41.3a	2,339.0a	46.3a	970.1a	28.7a	33.8a	2034.1a	47.7a
		S2	1,040.8a	25.2a	41.3a	2,222.9a	46.8a	966.7a	28.6a	33.8a	2027.8a	47.7a
	D3	CK	1,039.1a	25.1a	41.4a	2,230.4a	46.6a	951.4a	27.9a	34.1a	1994.4a	47.7a
		S1	1,034.9a	24.7a	41.9a	2,247.3a	46.1a	931.6a	27.4a	34.0a	1990.9a	46.8a
		S2	928.2b	22.1b	42.0a	1986.7b	46.8a	810.5b	23.7b	34.2a	1721.1b	47.1a
	D5	CK	1,039.1a	25.1a	41.4a	2,230.4a	46.6a	951.4a	27.9a	34.1a	1994.4a	47.7a
		S1	948.5b	22.8b	41.6a	2050.4b	46.3a	835.5b	24.5b	34.1a	1806.1b	46.3a
		S2	867.6c	21.8b	39.8b	1912.3c	45.3b	782.1c	23.7b	33.0b	1753.0c	44.6b
	D7	CK	1,039.1a	25.1a	41.4a	2,230.4a	46.6a	951.4a	27.9a	34.1a	1994.4a	47.7a
		S1	886.2b	21.2b	41.8a	1933.6b	45.9a	801.5b	23.3b	34.4a	1721.1b	46.6a
		S2	664.3c	17.3c	38.4b	1508.6c	44.0b	577.8c	18.0c	32.1b	1593.6c	36.3b
Kenong9204	D1	CK	977.6a	23.5a	41.6a	2007.9a	48.7a	858.8a	22.7a	37.8a	1784.8a	48.1a
		S1	819.3b	19.6b	41.8a	1742.3b	47.0b	758.0b	20.0b	37.9a	1604.2b	47.3a
		S2	772.3c	18.3c	42.2a	1721.1b	44.9c	672.0c	17.5c	38.4a	1,476.7c	45.5b
	D3	CK	977.6a	23.5a	41.6a	2007.9a	48.7a	858.8a	22.7a	37.8a	1784.8a	48.1a
		S1	690.4b	16.4b	42.1a	1,540.5b	44.8b	636.3b	16.7b	38.1a	1,444.9b	44.0b
		S2	497.2c	12.4c	40.1b	1519.2c	32.7c	414.4c	11.2c	37.0b	1,168.6c	35.5c
	D5	CK	977.6a	23.5a	41.6a	2007.9a	48.7a	858.8a	22.7a	37.8a	1784.8a	48.1a
		S1	633.7b	14.5b	43.7a	1,402.4b	45.2b	550.2b	14.0b	39.3a	1,232.4b	44.6b
		S2	172.7c	5.5c	31.4b	1,179.3c	14.6c	346.1c	11.2c	30.9b	1,168.6c	29.6c
	D7	CK	977.6a	23.5a	41.6a	2007.9a	48.7a	858.8a	22.7a	37.8a	1784.8a	48.1a
		S1	521.3b	12.5b	41.7a	1,306.8b	39.9b	448.4b	11.8b	38.0a	1,083.6b	41.4b
		S2	98.7c	3.5c	28.2b	1,104.9c	8.9c	267.3c	9.0c	29.7b	1,030.5c	25.9c
ANOVA	Cultivars (Cul)		**	**	**	**	**	**	**	**	**	**
	Duration (D)		**	**	**	**	**	**	**	**	**	**
	Intensity (In)		**	**	**	**	**	**	**	**	**	**
	Cul × D		**	**	**	**	**	**	**	**	**	**
	Cul × In		**	**	**	**	**	**	*	**	NS	**
	D × In		**	**	**	**	**	**	**	**	**	**
	Cul × D × In		**	**	**	**	**	**	**	**	NS	**

Different letters indicate significant differences (*P* < 0.05) among the treatments in each duration.

*NS* not significant.

*Significant at *P* < 0.05 level; **significant at *P* < 0.01 level. CK, control with 100% of natural light; S1, 40% of natural light; S2, 10% of natural light; D1, 1 day; D3, 3 days; D5, 5 days; D7, 7 days.

According to Path Analysis, grain number is the main influence factor to grain yield (Table 3). The direct effect value of grain number on grain yield in Henong825 and Kenong9204 was 0.875 and 0.861, respectively.

**Table 3:** The path coefficient analysis showing the direct and indirect effects of yield components on the grain yield of both cultivars.

Cultivars	Variables	Path coefficient		Total correlation
		Direct effect	Indirect effect	
Henong825	Grain number	0.875	− 0.140	0.735
	Grain weight	0.669	− 0.184	0.485
Kenong9204	Grain number	0.861	0.127	0.988
	Grain weight	0.182	0.600	0.782

### Changes in photosynthetic parameters under shading

#### Pn

**Figure 2:**
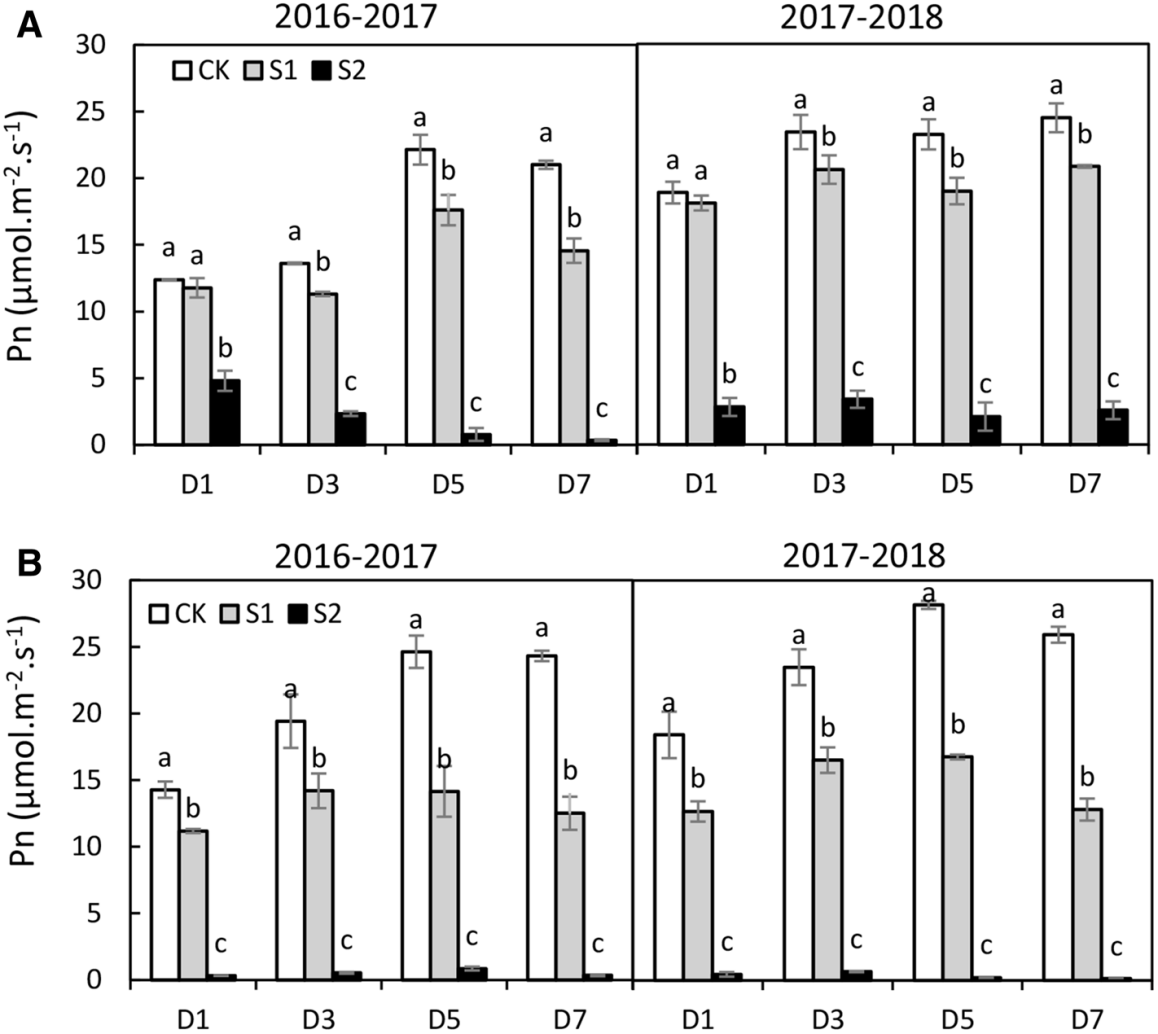
The photosynthetic rate (Pn) of flag leaf under different shading treatments in 2016–2017 and 2017–2018. Values followed by the different letter within each duration indicate significant difference (LSD, *P* < 0.05). (**A**) Henong825; (**B**) Kenong9204; CK, control with 100% of natural light; S1, 40% of natural light; S2, 10% of natural light; D1, 1 day; D3, 3 days; D5, 5 days; D7, 7 days.

Shading stress seriously reduced the Pn of both cultivars. The decline degree in S2 was much greater than that incurred in S1 (Fig. 2). Moreover, the impact of shading stress on the Pn of Kenong9204 was worse than it was on that of Henong825. The Pn of Henong825 was attenuated by 4.5% and 73.1% in D1, by 14.4% and 84.2% in D3, by 19.4% and 93.7% in D5, and by 22.8% and 93.9% in D7 in the S1 and S2 respectively (Fig. 2A). Whereas that of Kenong9204 was declined by 26.4% and 97.6% in D1, by 28.3% and 97.2% in D3, by 41.5% and 97.9% in D5, and by 49.6% and 99.0% in D7 correspondingly (Fig. 2B).

Pn rebounded quickly but differently among shading intensity and duration in both cultivars on the next day after shading removal (Supplementary Fig. S1). The Pn of Henong825 was significantly higher than and similar to that in the control under S1 and S2 respectively (Supplementary Fig. S1A). However, the Pn of Kenong9204 was still significantly lower than that of control in D5 and D7 (Supplementary Fig. S1B).

#### Stomata

**Figure 3:**
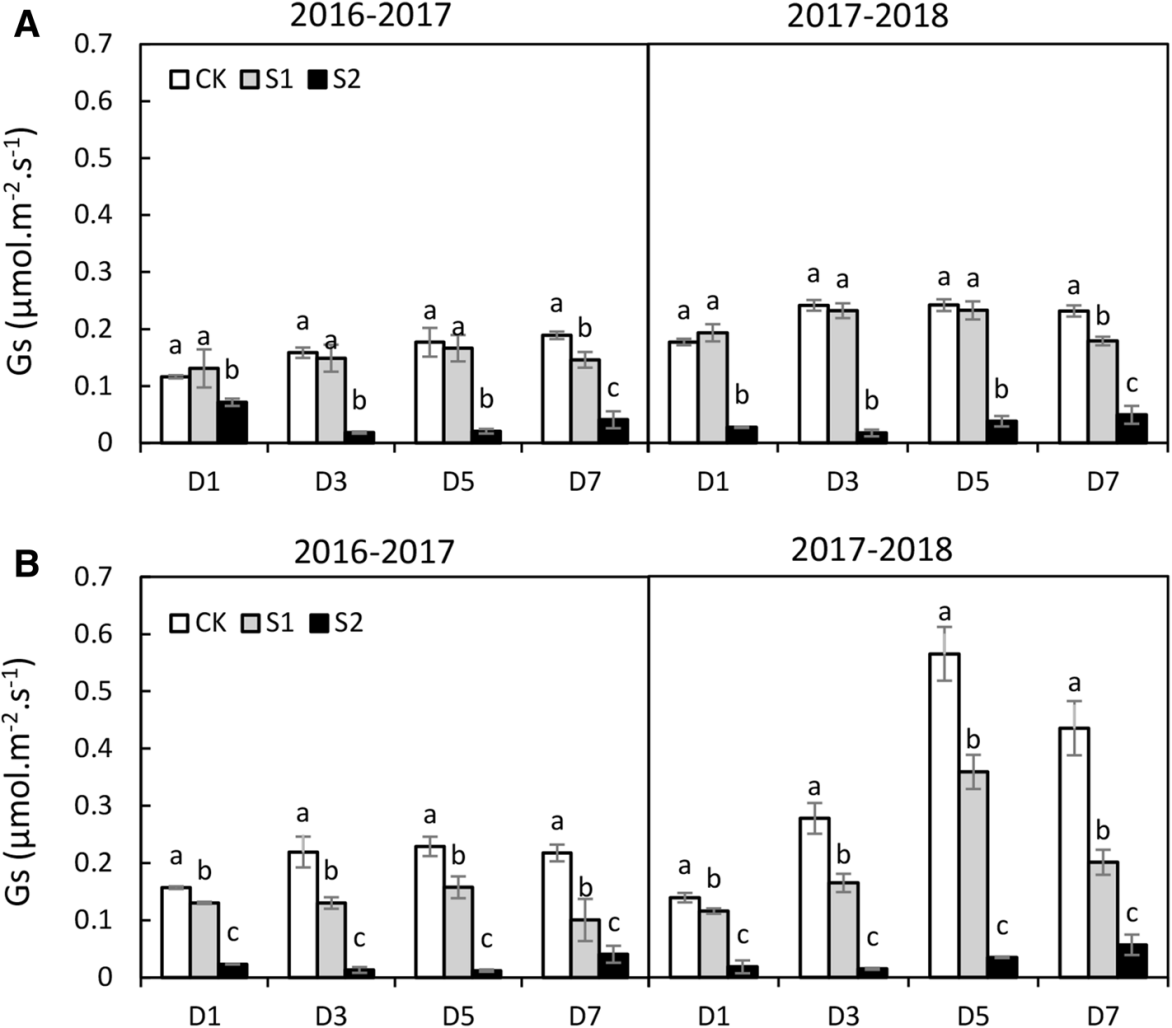
The stomatal conductance (Gs) of flag leaf under different shading treatments in 2016–2017 and 2017–2018. Values followed by the different letter within each duration indicate significant difference (LSD, *P* < 0.05). (**A**) Henong825; (**B**) Kenong9204; CK, control with 100% of natural light; S1, 40% of natural light; S2, 10% of natural light; D1, 1 day; D3, 3 days; D5, 5 days; D7, 7 days.

**Figure 4:**
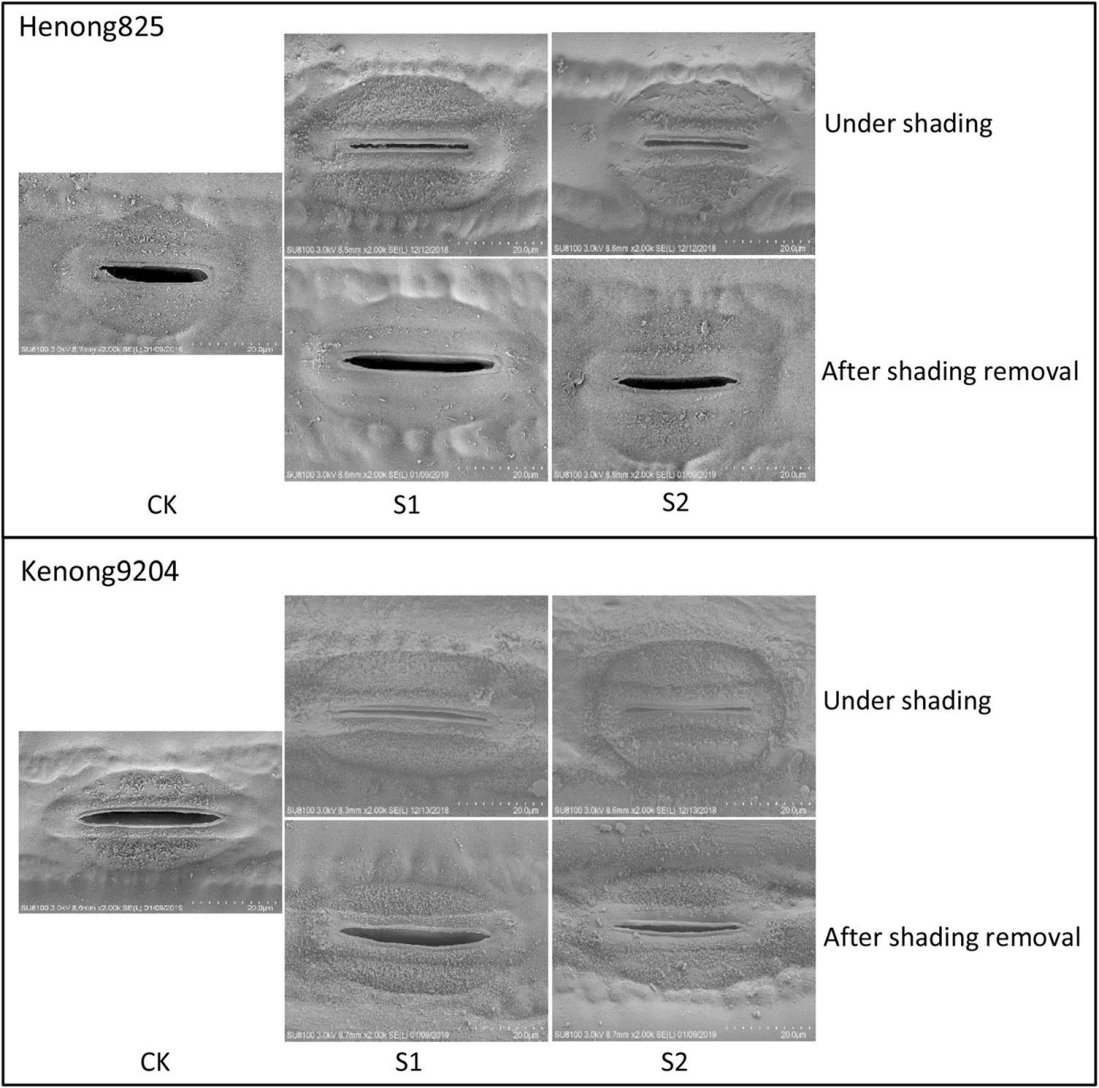
The electron micrographs of the stomata of Henong825 and Kenong9204 observed in different shading intensity for 7 days. CK, control with 100% of natural light; S1, 40% of natural light; S2, 10% of natural light; under shading, shading period; after shading removal, shading removal after one day; Bars = 20 μm.

The Gs of both cultivars decreased as the shading intensity and duration increased, and a lower Gs was observed in S2 than in S1 (Fig. 3). Moreover, the Gs of Kenong9204 was more sensitive to shading than that of Henong825. The Gs of Henong825 was not significantly affected by S1, except for D7 (Fig. 3A). It was extensively decreased by 61.6–90.7% under S2. The Gs of Kenong9204 was seriously reduced by 16.9–53.8% and 84.1–94.3% under S1 and S2 (Fig. 3B). The closure of stomatal aperture was also observed after 7 days exposure to S1 and S2 (Fig. 4). The stomata of Kenong9204 almost closed in response to S2. On the next day after shading removal, the stomatal aperture size of both cultivars augmented. Henong825 showed higher resilience than Kenong9204.

The intercellular CO_2_ concentration (Ci) of both cultivars increased as the shading intensity and duration increased (Supplementary Fig. S2). The higher Ci was observed in S2 than in S1. In D1 and D3, the Ci of Henong825 was lower than that of control, and significantly higher in S2 (Supplementary Fig. S2A). Under D5 and D7, the Ci of Henong825 was increased by 14.4% and 34.5% in S1, by 96.5% and 129.0% in S2. For Kenong9204, the Ci was increased by 31.2–117.9% in S1, by 59.5–192.7% in S2 (Supplementary Fig. S2B).

#### Chlorophyll content, leaf anatomy and chloroplast ultrastructure

**Figure 5:**
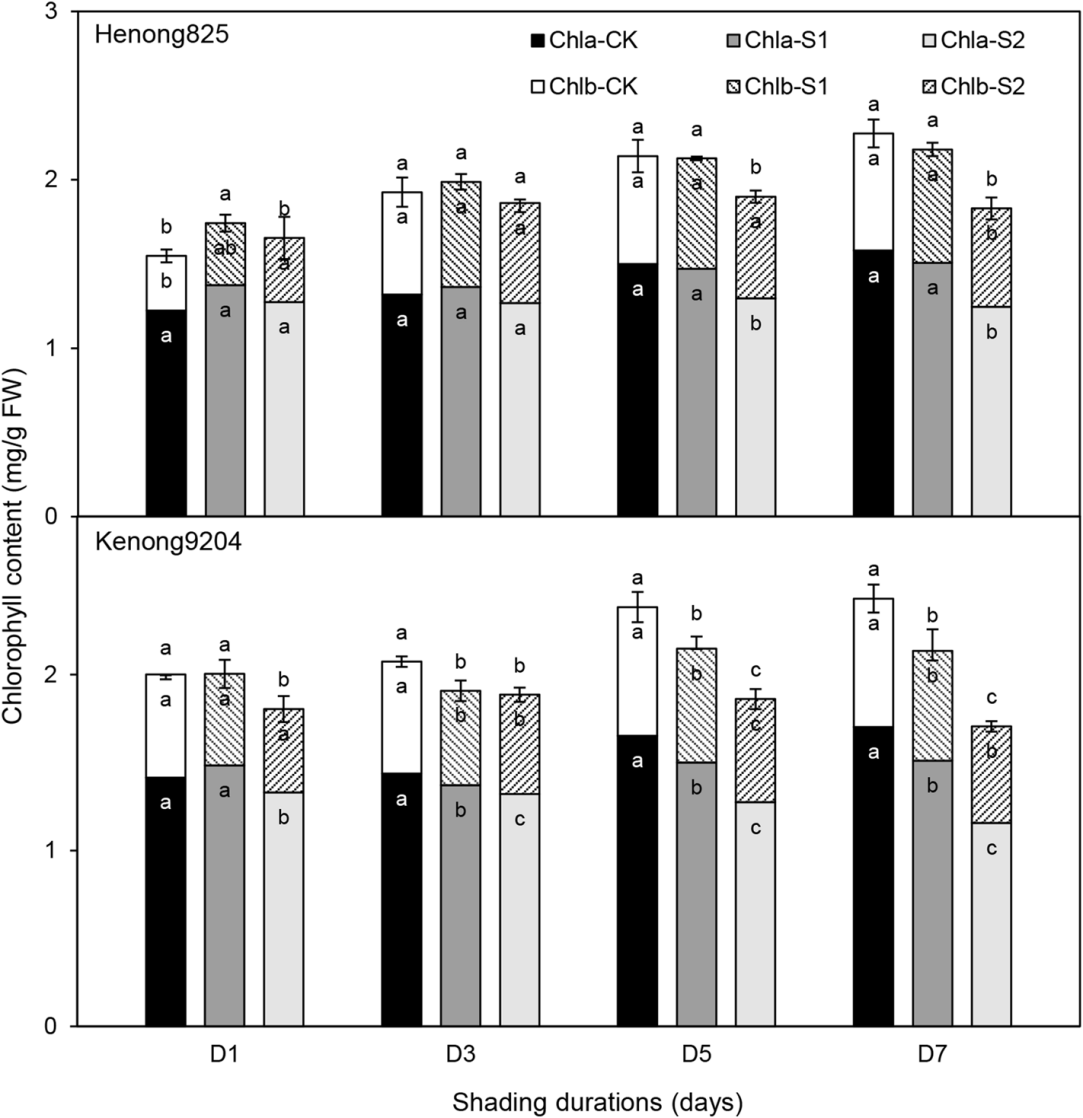
The chlorophyll *a* (chl *a*), chlorophyll *b* (chl *b*), and chlorophyll *a* + *b* (chl *a* + chl *b*) contents of the flag leaves of Henong825 and Kenong9204 under different shading treatments. Values followed by the different letter within each duration indicate significant difference (LSD, *P* < 0.05). CK, control with 100% of natural light; S1, 40% of natural light; S2, 10% of natural light; D1, 1 day; D3, 3 days; D5, 5 days; D7, 7 days.

With shading intensity and duration increasing, the chlorophyll content of both cultivars decreased (Fig. 5). In the same shading period, the chlorophyll content of both cultivars in S1 was markedly higher than that in S2. The reduction of chlorophyll content was due to the reduction of chlorophyll a (chl a) and chlorophyll b (chl b). However, the extent of shading effect in both cultivars was different. The chlorophyll content of Henong825 was not significantly affected in S1, but significantly increased in S1–D1. The chl a and chl b of Henong825 were not significantly affected in S1. Under S2, the chlorophyll content of Henong825 significantly decreased in D5 and D7, except in D1 and D3. The chl b of Henong825 significantly increased in S2–D1, and significantly decreased in S2–D7. For Kenong9204, the chlorophyll content significantly decreased in S1 and S2, but not in S1–D1.

**Figure 6:**
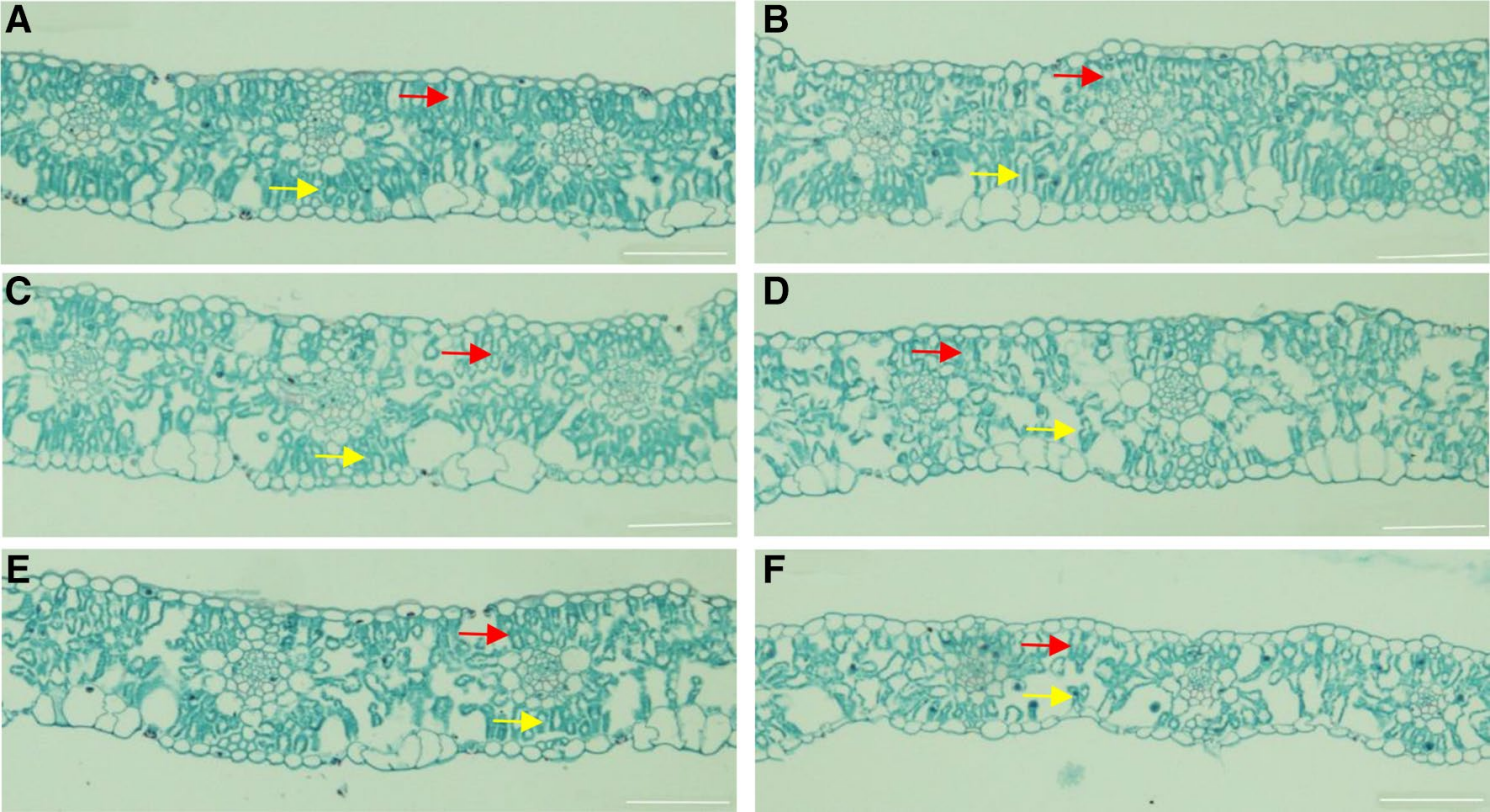
The leaf anatomical structure of Henong825 (**A**, **C**, **E**) and Kenong9204 (**B**, **D**, **F**) under different shading intensity for 7 days. (**A**, **B**) Control with 100% of natural light (CK); (**C**, **D**) 40% of natural light (S1); (**E**, **F**) 10% of natural light (S2); yellow arrows are palisade tissue, red arrows are spongy parenchyma; bars = 100 μm.

In terms of leaf microstructure, shading adversely affected the anatomy of flag leaf (Fig. 6). Compared to control, leaf under D7 thinned, and so did the palisade tissue and spongy tissue. The compact palisade tissue and spongy tissue were disrupted, loose, and irregularly arranged. The effect degree of leaf in S2 was much greater than that in S1. The effects of shading on the anatomy of flag leaf were more pronounced in Kenong9204 than in Henong825.

**Figure 7:**
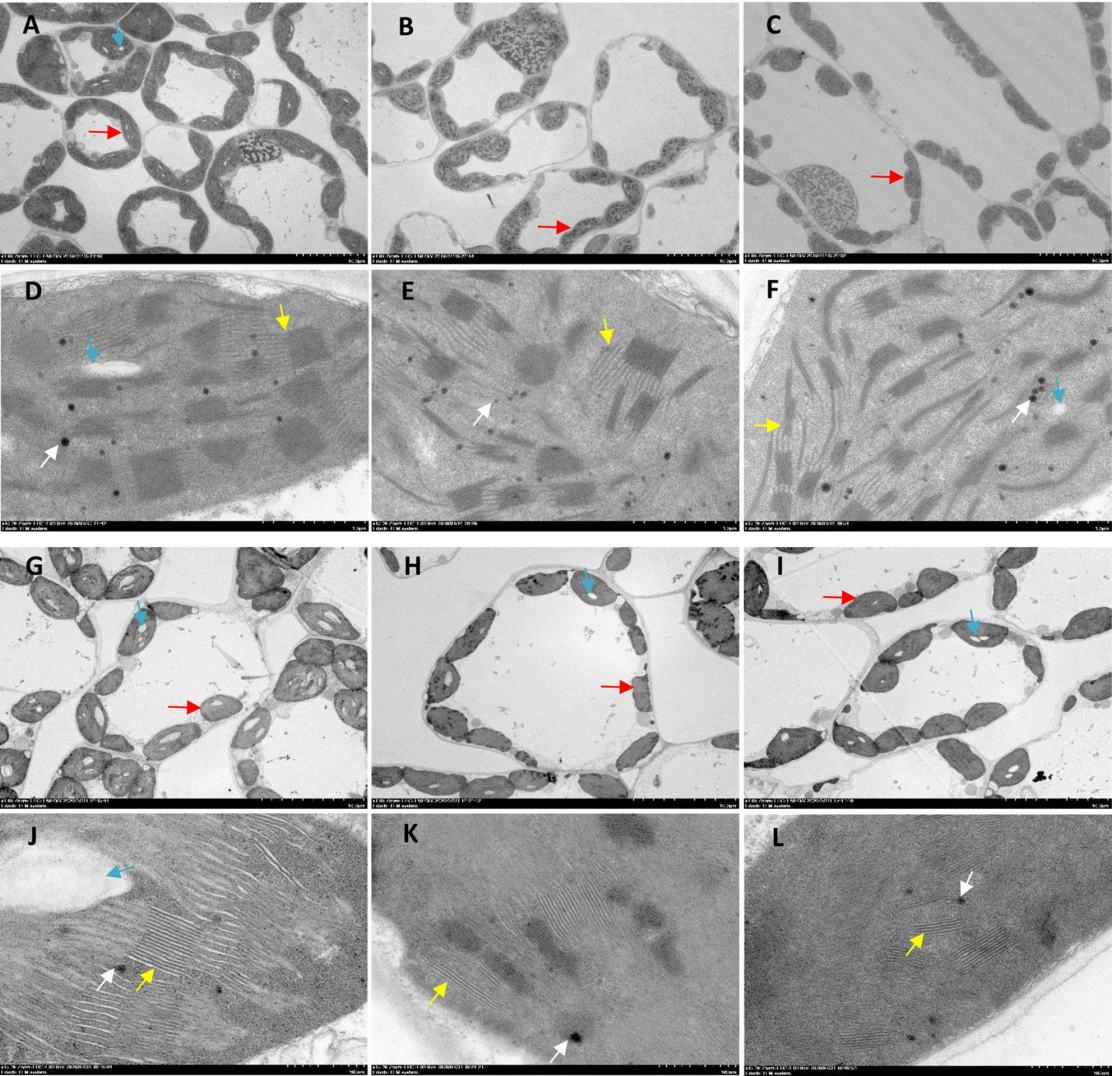
The electron micrographs of flag leaf chloroplasts in Henong825 (**A**–**F**) and Kenong9204 (**G**–**L**) under different shading intensity for 7 days. (**A**, **D**, **G**, **J**) Control with 100% of natural light (CK); (**B**, **E**, **H**, **K**) 40% of natural light (S1); (**C**, **F**, **I**, **L)** 10% of natural light (S2); red arrows are chloroplasts, blue arrows are starch grains, yellow arrows are chloroplast grana, white arrows are osmiophilic particles. (**A**–**C**, **G**–**I**) bar = 10 μm, (**D**–**F**, **J**–**L**) bars = 1 μm.

Compared to control, the number and size of chloroplasts decreased in different shading intensities for D7 in both cultivars (Fig. 7). And the stronger shade intensity was, the greater the chloroplasts was affected by shading. The number of starch granules was lower and their size was smaller in the chloroplast. The grana lamellae was reduced and thinned, especially for Kenong9204. The number of osmiophilic particles of Henong825 increased, whereas Kenong9204 did not.

#### Chlorophyll fluorescence parameters

Shading decreased Fv/Fm, the YII, Jmax and PARsat of leaf in wheat, with shading intensity and duration increased (Table 4). The responses of Kenong9204 was sensitive to shading stress more than Henong825. For Kenong9204, Fv/Fm, the YII, Jmax and PARsat were significantly reduced from D1 to D7, except Fv/Fm in S1–D1. For Henong825, the Fv/Fm was not significantly affected in S1, and only significantly decreased in S2–D3 and S2–D7. Under D1 and D3, the YII of Henong825 was not significantly damaged in two shading intensity, and significantly increased in S1–D1. Under D5 and D7, the YII of Henong825 showed a significant decrease in both shading intensity, except in S1–D5. The Jmax and PARsat of Henong825 were significantly reduced in response to shading stress, but not in S1–D1. Under S1 and S2, the Jmax was reduced by 1.4–16.8% and 19.2–28.8% in Henong825, by 10.1–19.5% and 26.0–33.1% in Kenong9204.

**Table 4:** The effects of shading on the chlorophyll fluorescence parameters of flag leaf in 2016–2017 and 2017–2018.

Cultivars	Shading treatments		2016–2017				2017–2018			
	Durations	Intensities	Fv/Fm	Y(II)	Jmax	PARsat	Fv/Fm	Y(II)	Jmax	PARsat
Henong825	D1	CK	0.835a	0.653b	72.0a	5.88a	0.805a	0.503b	82.3a	6.81a
		S1	0.838a	0.688a	71.8a	5.74a	0.804a	0.533a	80.2a	6.63a
		S2	0.834a	0.651b	56.3b	4.83b	0.800a	0.503b	68.6b	5.47b
	D3	CK	0.841a	0.516a	72.5a	7.55a	0.811a	0.537a	72.0a	6.26a
		S1	0.840a	0.511a	65.2b	6.18b	0.809a	0.535a	59.1b	5.89b
		S2	0.826b	0.498a	53.8c	5.15c	0.790b	0.515a	49.0c	5.25c
	D5	CK	0.843a	0.621a	68.0a	5.88a	0.814a	0.533a	81.2a	6.63a
		S1	0.836a	0.604ab	54.0b	5.37b	0.810a	0.516a	70.7b	6.38b
		S2	0.820b	0.582b	48.9c	4.74c	0.780b	0.474b	63.3c	5.75c
	D7	CK	0.839a	0.629a	77.2a	8.34a	0.812a	0.492a	80.2a	6.52a
		S1	0.831a	0.604b	63.4b	6.05b	0.805a	0.476b	72.2b	6.23b
		S2	0.802b	0.585c	50.6c	4.46c	0.764b	0.436c	61.7c	5.75c
Kenong9204	D1	CK	0.829a	0.574a	76.1a	7.89a	0.811a	0.486a	74.5a	6.47a
		S1	0.826a	0.550b	58.6b	5.86b	0.806a	0.470b	64.6b	5.88b
		S2	0.818b	0.538c	44.4c	4.20c	0.798b	0.435c	56.4c	5.38c
	D3	CK	0.835a	0.624a	68.9a	6.57a	0.825a	0.530a	76.4a	6.31a
		S1	0.824b	0.600b	52.5b	5.46b	0.801b	0.512b	68.4b	5.66b
		S2	0.817c	0.585c	48.1b	5.00c	0.787c	0.472c	54.3c	5.24c
	D5	CK	0.836a	0.622a	62.7a	5.74a	0.835a	0.513a	78.9a	6.19a
		S1	0.813b	0.596b	55.8b	5.05b	0.797b	0.489b	71.6b	5.98b
		S2	0.778c	0.569c	48.5c	4.49c	0.777c	0.432c	55.7c	5.29c
	D7	CK	0.845a	0.626a	70.3a	6.28a	0.825a	0.503a	88.4a	7.67a
		S1	0.811b	0.600b	57.0b	5.44b	0.794b	0.464b	70.6b	6.26b
		S2	0.775c	0.569c	43.5c	4.49c	0.764c	0.419c	63.5c	5.69c
ANOVA	Cultivar (Cul)		**	*	**	**	*	**	**	**
	Duration (D)		**	**	**	**	**	**	**	**
	Intensity (In)		**	**	**	**	**	**	**	**
	Cul × D		**	**	**	**	NS	**	**	**
	Cul × In		**	**	**	*	**	*	**	**
	D × In		**	*	**	**	**	*	**	**
	Cul × D × In		**	NS	**	**	*	*	**	**

Different letters indicate significant differences (*P* < 0.05) among the different shading treatments. CK, control with 100% of natural light; S1, 40% of natural light; S2, 10% of natural light; D1, 1 day; D3, 3 days; D5, 5 days; D7, 7 days; Fv/Fm, maximal photochemical efficiency of photosystem II; YII, actual photochemical quantum efficiency; Jmax, maximum electron transport; PARsat, saturation irradiance.

*NS* not significant.

*Significant at the *P* < 0.05 level; **Significant at the *P* < 0.01 level.

## Discussion

Shading, which causes lower PAR, is an important environmental stressor that constrains wheat development and yield formation^13,33^. In the early reproductive stage of wheat, such as the YM stage, shading has a great impact on grain formation. Shading stress may limit carbohydrate availability^13^ and it may augment the number of sterility spikelets^17^. Finally, the grain number per ear decreased significantly. In this study, shading during YM stage caused grain yield reduction of shade-sensitive cultivar by 14.0–47.2% under 60% shading and 21.4–79.4% under 90% shading (Table 2), which was strongly related to grain number decrement (Table 3). The present study lent great supports to the above mentioned findings. Furthermore, with shading duration and intensity increasing, grain number in both cultivars significantly decreased. For shade-tolerant cultivar, Henong825, shading shorter than 3 days had no effect on grain number under 60% shading. Under 7 days of shading 90%, the grain number of Henong825 was decreased by 33.3%. For shade-sensitive cultivar, Kenong9204, the grain number was decreased by 14.2% under 1 day of 60% shading, and it was deceased by 72.7% under 7 days of 90% shading. This result confirmed our supposition that shade-sensitive cultivar suffer more from shading stress.

With shading duration increasing, a source limitation in the reproductive stage may restrain the sink strength such as grain number^34,35^, which may have reduced the redistribution of stored dry matter from vegetative organs to grains after shading removal. Shading stress decreases the Pn of plants, affects the soluble carbohydrates available for transport to the spike of crops^36^, limits grain formation, and ultimately reduces grain yield. In this study, the Pn of both cultivars decreased with the shading duration and intensity. However, different cultivars responded differently to shading. The Kenong9204 was more sensitive to shading than Henong825. Under S1, the leaf Pn in Henong825 during YM stage was not significantly impacted in D1, which was mainly due to increased Gs. Under S1–D3 and S1–D5, the reduction of Pn in shading stress could be primarily attributed to the reduction of solar radiation in the shading treatments. Huang et al.^30^ concluded that the reduction of the Pn under shading was related to the decline of Gs. In this study, with shading intensity and duration increasing, the leaf Gs was decreased, while the Ci increased under shading. This indicated that the reduction of Pn was mainly related to low solar radiation as well as the non-stomatal restriction, especially under 90% of shading condition. Chlorophyll content is an essential factor in determining Pn^37^. In the present study, the chlorophyll content of leaves under shading conditions was less than that in control, and the reduction of chl *a* was faster than that of chl *b*. This showing that shading stress impaired their photosynthetic machinery. Chlorophyll fluorescence can accurately reflect photosynthetic change and plant responses to the environment^38,39^. Fv/Fm is the maximal photochemical efficiency of photosystem II, which reflecting the capacity of solar energy use in PSII. YII is the actual photochemical quantum efficiency when leaf is in the light. In this study, shading reduced Fv/Fm and YII, as well as lessened Jmax and PARsat. These findings suggest that shading can impair the photosynthetic system center, leading to the usage of solar energy capacity in PSII and reducing the actual light energy conversion efficiency. The reduction in the quantity of electrons passing through PSII ultimately negatively affected photosynthesis.

In addition, the leaf, was affected under shading conditions, consistent with the observations of Hussain^40^. Considering the photosynthetic performance of plants^41^, the ultrastructure of chloroplasts may have been altered with shading conditions, thereby affecting photosynthesis. Under longer shading duration (longer than 5 days), the thickness of their leaves, palisade tissue, and spongy tissue significantly decreased, resulting in the reduction of their photosynthetic area. And the shade-tolerant cultivar was less impact by shading than the shade-sensitive cultivar. Thus, the Pn rebounded quickly in shade-tolerant cultivar, but it did not in shade-sensitive cultivar.

Furthermore, the low radiation due to net shading on the top of canopy was accompanied by the change in spectral fractions, corroborating the findings of Li et al.^10^. This fraction of the visible light in the S2 treatments may have altered leaf photosynthesis; nonetheless, further studies are needed. Additionally, a < 1 °C shift in air temperature and < 5% change relative humidity with shading conditions may cause only a marginal effect in crop development, compared to the impact of radiation reduction^42^. Thus, it was indeed the reduction of light intensity that most significantly affected wheat development with shading conditions.

## Conclusion

**Figure 8:**
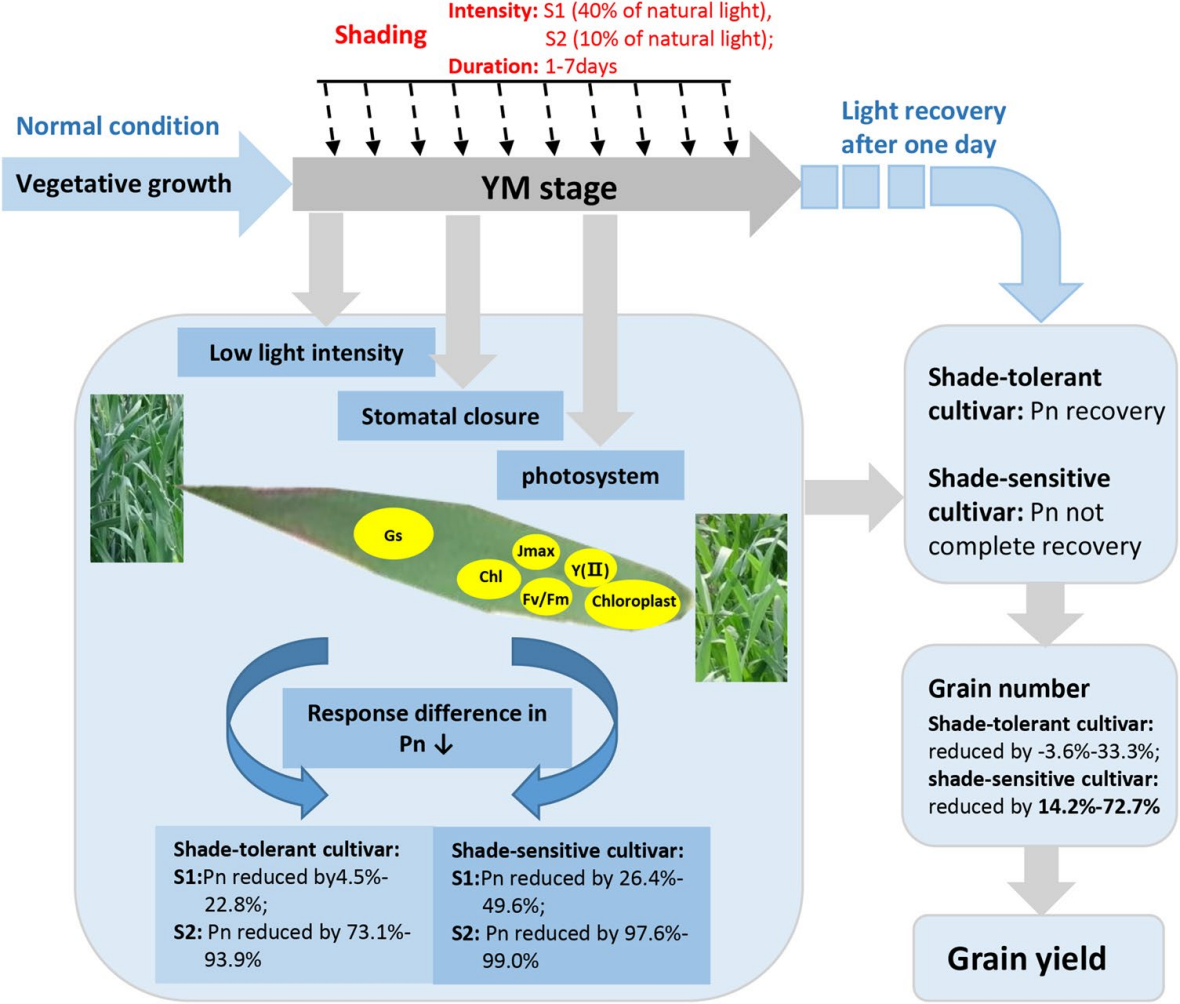
A model of wheat grown under low light. The downward black arrow indicates a descent.

To draw a conclusion, low light stress, during YM stage decreased wheat grain yield, mainly due to the reduction of grain number (Fig. 8). The reduction of grain number was related to the leaf Pn. Under moderate (60% shading) and short shading (shorter than 3 days), the reduction of leaf Pn in the shade-tolerant cultivar was mainly related to low light energy. With shading intensity and duration increasing, the photosynthetic system was impaired, chlorophyll content dropped, Gs decreased, photosynthetic system damaged. This indicated that the reduction of leaf Pn was not only related to low light, but also related to damage of the leaf photosynthetic system, which was more obvious in the shade-sensitive cultivar (Kenong9204).

## Materials and methods

### Wheat cultivars and growing conditions

In this study, pot and field experiments with the shade-tolerant cultivar Henong825 and the shade-sensitive cultivar Kenong9204 were performed. These two winter wheat cultivars were identified with different degrees of shade tolerance by our previous study^17^. Both cultivars are released by Hebei Province, China, which are the most widely planted wheat cultivars in North China Plain. The parental combination of Henong825 and Kenong9204 is Linyuan95-3091/Shi4185, SA502/6021, respectively. Henong825 is characterized by strong lodging resistance. Kenong9204 is characterized by suitable for moderate water and fertilizer. Two field experiments were conducted during the 2016–2017 and 2017–2018 wheat-growing seasons in the Luancheng agro-ecosystem experimental station of the Chinese Academy of Sciences, Hebei Province (37° 53′ N and 114° 41′ E; elevation at 50 m). The climate characterizing of the study region is summer monsoon. The mean temperature, total precipitation, and solar radiation in both the winter wheat-growing seasons are shown in Table 5. The soil used in the experiments was loam containing 21.41 g kg^−1^ organic matter, 109.55 mg kg^−1^ alkaline nitrogen (N), 1.44 g kg^−1^ total N, 15.58 mg kg^−1^ available phosphorus (P), and 220 mg kg^−1^ rapidly available potassium (K). In both seasons, soils were fertilized with urea (N, 46%) and complete fertilizer (N–P, 21–54%) at 300 kg ha^−1^ and 375 kg ha^−1^. Seeds were sown by hand on October 6, 2016 and October 17, 2017, then the seedlings emerged 1 week later. In 2017 growing season, the YM stage was on April 15, and anthesis stage was on May 1 in both cultivars. In 2018 growing season, the YM stage was on April 16, and anthesis stage was on May 2 in both cultivars. The seedling density was 166 m^−2^, which is the norm in this region.

**Table 5:** The monthly mean temperature (°C), total precipitation (mm), and solar radiation (MJ m^−2^ day^−1^) during the two growing seasons of winter wheat in 2016–2017 and 2017–2018.

Month	Mean temperature (°C)		Precipitation (mm)		Solar radiation (MJ m^−2^ day^−1^)	
	2016–2017	2017–2018	2016–2017	2017–2018	2016–2017	2017–2018
October	13.1	12.4	44.8	8.4	10.4	7.8
November	5.0	5.2	3.2	0.6	9.0	8.9
December	0.0	− 0.3	4.0	0.5	6.7	8.3
January	− 1.9	− 2.6	1.1	0.9	6.7	6.6
February	2.4	0.7	5.7	0.0	10.6	11.1
March	8.5	9.4	4.8	7.1	14.0	13.1
April	16.0	15.5	19.0	66.3	17.8	16.5
May	22.4	21.4	16.4	50.3	23.1	19.3
June	23.8	26.2	8.7	23.2	21.1	21.9

### Experimental design

This study was a combination of field experiment and pot experiment to investigate the effect of different shading intensity and duration during YM stage on grain components and photosynthetic characteristics. Pot experiment was supplement to field experiment.

#### Field experiments

The experiments were arranged in a randomized split-split plot design with three replicates. The main plots were split into three subplots subjected to one of three shading intensities: 100% (CK, control), 40% (S1), and 10% (S2) of natural light. Each subplot was split into four sub-subplots, which were randomly allocated to one of four durations: 1 day (D1), 3 days (D3), 5 days (D5), and 7 days (D7) during the YM stage. The shading treatments were conducted in these periods and replicated three times. Each plot size was 6 m long and 2 m wide, with 40 rows. There were 72 plots. Different degrees of artificial shade were provided by using black polyethylene screens horizontally installed at a height of 2 m above the ground.

#### Determination of YM stage

**Figure 9:**
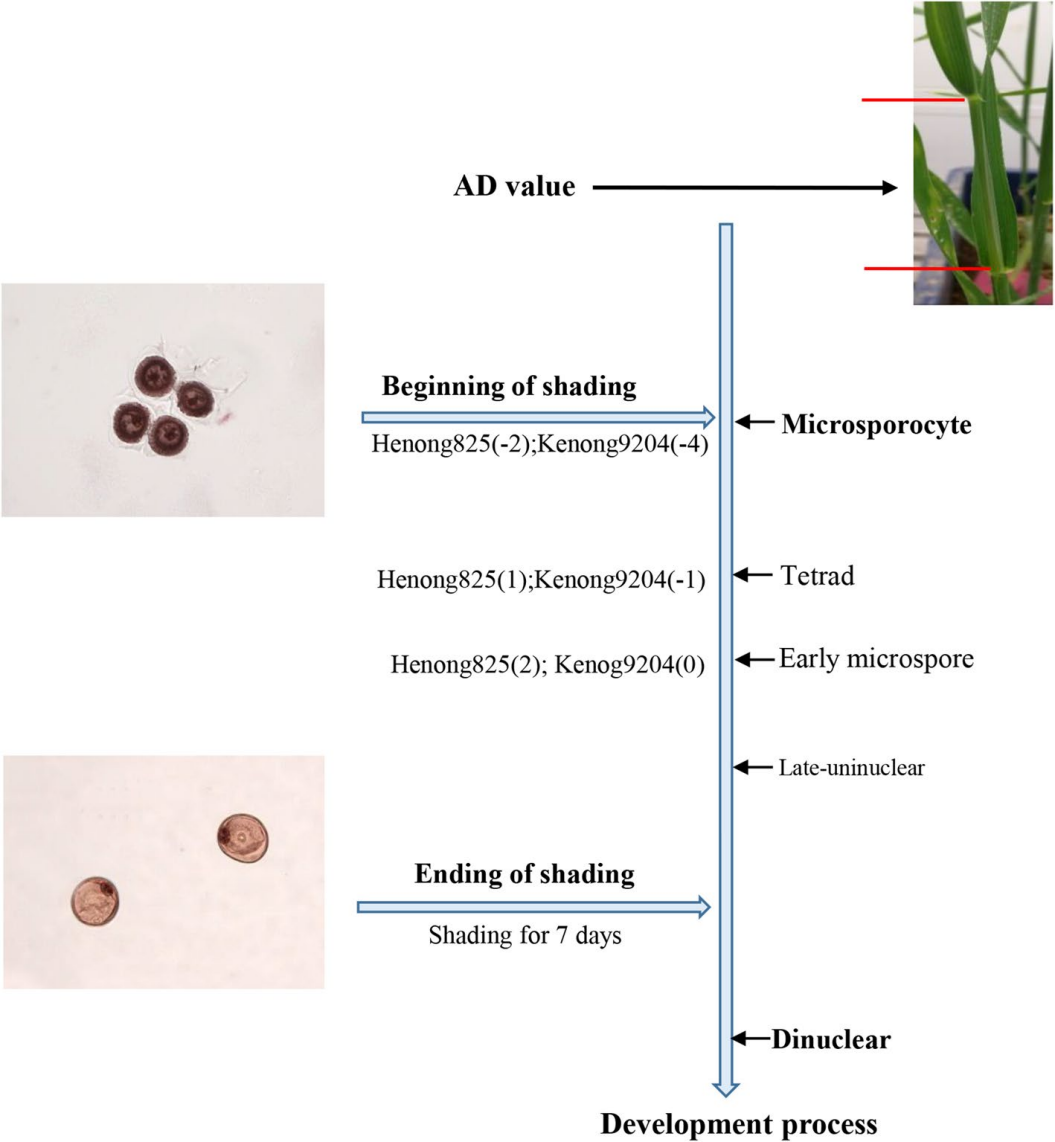
The relationship between anther development and shading period in two wheat cultivars.

The YM stage roughly corresponds to Zadok’s scale from Z37 (main stem with flag leaf is visible) to Z39 (flag leaf ligule is noticeable). According to previous researches of YM stage, the estimated measurement of the YM stage was based on the auricle distance (AD, the distance between the auricle of the flag leaf and the auricle of the penultimate leaf) of main stem^43,44^. In order to keep the relationship between the occurrence of YM and AD unchanged, the field management practices, adequate irrigation was the same in two growing-seasons. Moreover, for each experiment, at the onset of appearance of the flag leaf of the main stem, 30 anthers of ten main stem spike of wheat were randomly sampled to establish the timing of YM stage initiation^1^. The correlation of the AD with the development of the YM stage in the florets of the two cultivars was measured and observed using microscope (Fig. 9). The cultivar Henong825 reached the YM stage at 1–2 cm, whereas Kenong9204 reached the YM stage at − 1 to 0 cm. To capture the YM stage in the shading condition, the plants were subjected to shading stress ahead of the YM stage occurrence. When more than 50% of the plants in each plot reached − 2 cm in Henong825 and − 4 cm in Kenong9204, the main stem of the plants was tagged, and shading stress was applied in each plot. Each experimental plot for Henong825 and Kenong9204 was independently subjected to shading stress on April 15, 2017 and April 16, 2018. When the shading stress treatments ended, the shade screens were removed and were exposed to natural light until they matured. Air temperature, light intensity, and relative humidity above the canopy were recorded using a portable weather station (ECA-YW0501; Beijing, China) during the shading period. Light spectral was measured using a portable geographic spectrometer (PSR + 3500, USA). The irradiance of spectral wavelength ranging from 350 to 2,500 nm was measured. The proportions of blue light (B/T), green light (G/T), red light (R/T), far-red light (FR/T), and red/far red (R/FR) were calculated according to their irradiance at 400–500 nm, 500–600 nm, 600–700 nm, and 700–800 nm, respectively. Following the local field management practices, adequate irrigation was conducted three times during the overwinter, jointing, and anthesis stages of the wheat-growing season. Weeds, fungal diseases, and insect pests were controlled through spraying of conventional herbicides, fungicides, and insecticides, correspondingly.

#### Pot experiments

The pot experiments were conducted in a temperature-controlled glasshouse. Vernalized seedlings of the two wheat cultivars were transplanted to pots (45 cm in length, 28.5 cm in width and 20 cm in height; 18 plants in each pot; three pots for each treatment group) containing a mixture of vermiculite and nutritional soil (1:1). All wheat seedlings were grown at a day temperature of 25 °C, night temperature of 15 °C, and light intensity of 800 μmol m^−2^ s^−1^. When the AD of the main stems of Henong825 and Kenong9204 cultivars were approximately − 2 cm and − 4 cm, respectively, the main stem of the plants was tagged, and shading stress was applied in each treatment. Shading treatments groups were the different shading intensities and shading durations previously mentioned. The shading condition in glasshouse was simulated with black polyethylene screen to keep up with the experimental methods in the field. After shading stress, the shading nets were removed, until the crops matured.

### Sampling and measurements

#### Photosynthetic rate, stomatal conductance, intercellular carbon dioxide, and chlorophyll fluorescence parameters

In field experiments, three randomly selected flag leaves on the tagged main stems of plants in each plot were analyzed to determine Pn, Gs, Ci, and chlorophyll fluorescence. For each shading treatment group, Pn, Gs, and Ci were measured using an LI-6400XT portable system (LI-COR Biosciences, Nebraska, USA), and the chamber of which was equipped with a red/blue LED light source (LI6400-02B) before the shading stress was removed. Before measurement, the machine was preheated for 30 min, and checked, adjusted to zero, calibrated according to the instructions. Moreover, the light intensity in measured chamber was equivalent of shading treatment conditions. The flow rates was set at 500 μmol s^−1^, The temperature in chamber was set 25 °C. The CO_2_ concentration was set to 400 μmol mol^−1^, which was provided by carbon dioxide cylinders to maintain a stable CO_2_ environment. The chlorophyll fluorescence of flag leaves on the tagged main stems of plants were measured using a modulate chlorophyll fluorescence imaging system (Imaging-PAM; Hansatech, UK) in each plot. The primary light energy conversion efficiency of PSII (Fv/Fm) and actual photochemical quantum efficiency (YII) were measured after 30 min of dark adaptation. The saturation irradiance (PARsat) and maximum electron transport (Jmax) of flag leaves in each treatment were calculated using a modified rectangular hyperbola. On the day next to shading removal, the Pn of three flag leaves from each replicate plot were measured.

#### Chlorophyll content

For glasshouse pot experiments, nine flag leaves (three leaves were randomly selected per pot from three pots in each treatment group) tagged main stems of plant were selected prior to the removal of shading. The flag leaves were then sliced following the removal of the main vein. After the sliced fresh leaves were weighed to 0.1 g, the chlorophyll content of leaves was extracted with 80% acetone for 48 h and analyzed through micro-determination (Thermo Varioskan Flash, USA). The absorbance of chlorophyll *a* (chl *a*) and chlorophyll *b* (chl *b*) was read at 663 and 646 nm, respectively (Thermo Varioskan Flash, USA), and the chlorophyll contents were calculated according to following equations: chl *a* (mg/g) = (12.7 × A663 nm–2.69 × A646 nm)/(100 × M); and chl b (mg/g) = (22.9 × A646 nm–4.68 × A663 nm)/(100 × M) where A663 and A646 are absorption levels at 663 and 646 nm, respectively; M is leaf fresh weight. The total chlorophyll (chl *a* + chl *b*) values were calculated by chl *a* and chl *b* values.

#### Leaf anatomy and surface characteristics

The approximately 2-mm^2^ leaf sections in D7 treatments and one day after recovery were harvested from the center of three flag leaves on the tagged main stems of plants using a scalpel and were rapidly fixed in electron microscope fixation fluid at 4 °C overnight. Stomatal apertures and chloroplast ultrastructure were observed by Servicebio (Wuhan) using a scanning electron microscope (SU8100; Hitachi) and a transmission electron microscope (HT7700; Hitachi). Simultaneously, the fully expanded flag leaves collected from plants in each treatment were fixed with FAA solution and embedded in paraffin to measure the leaf anatomical structure. The embedded wax block were sectioned to a thickness of 8 μm, then following dewaxing in environmental transparent solution and rehydration in a series of graded alcohol solutions. Finally, the tissue samples were stained with safranin and fast green, observed under a Leica DM6 microscope (Leica, Germany), and the respective images were obtained.

#### Grain yield, yield components, and aboveground biomass

At harvest in the field experiments during both growing seasons, 60 tagged plants per replicate were randomly sampled to determine grain yield components. The harvested plants were naturally dried to a grain water content of approximately 11%. Each tagged plant was then threshed using a single plant threshing machine to determine the grain number and grain yield needed for the estimation of the average grain weight. In addition, 30 tagged winter wheat plants were uprooted randomly and gradually by hand from each plot. Each plant was cut from the root and was dried at 80 °C. Aboveground biomass was measured using a precision digital balance (model BSA3202S; Sartorius, Germany) with a precision of 0.01 g.

### Statistical analysis

The experimental data for grain yield, yield components, biomass and chlorophyll fluorescence parameters were analyzed using a general linear model procedure (GLM) in SPSS 22.0 for a split-split plot design. The significant differences among treatment mean values were determined by the least significance difference analysis (LSD, *P* < 0.05). In addition, in order to better explanation of relationship between grain yield and yield components, the effects of grain components of grain yield were computed through the path coefficient analysis.

## Supplementary information

Supplementary Information 1

Supplementary Information 2
