# Supplementary figures and images for: Photosynthetic base of reduced grain yield by shading stress during the early reproductive stage of two wheat cultivars
Source: Sci Rep. 2020 Sep 1;10:14353. doi: 10.1038/s41598-020-71268-4 (PMC7463241; doi:10.1038/s41598-020-71268-4)

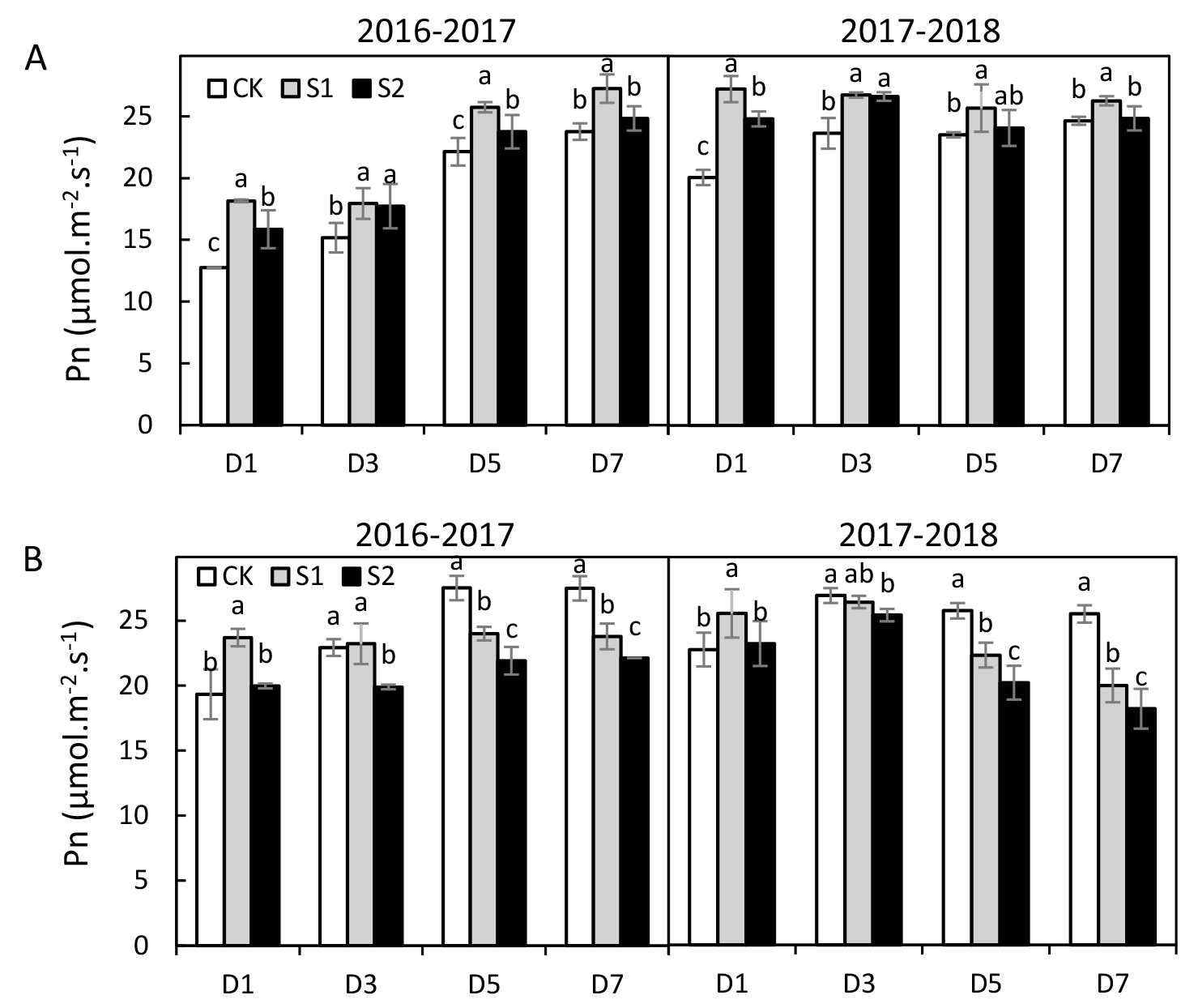

Supplement: Supplementary file 1 — Supplementary Information 1 [file 41598_2020_71268_MOESM1_ESM.tif]

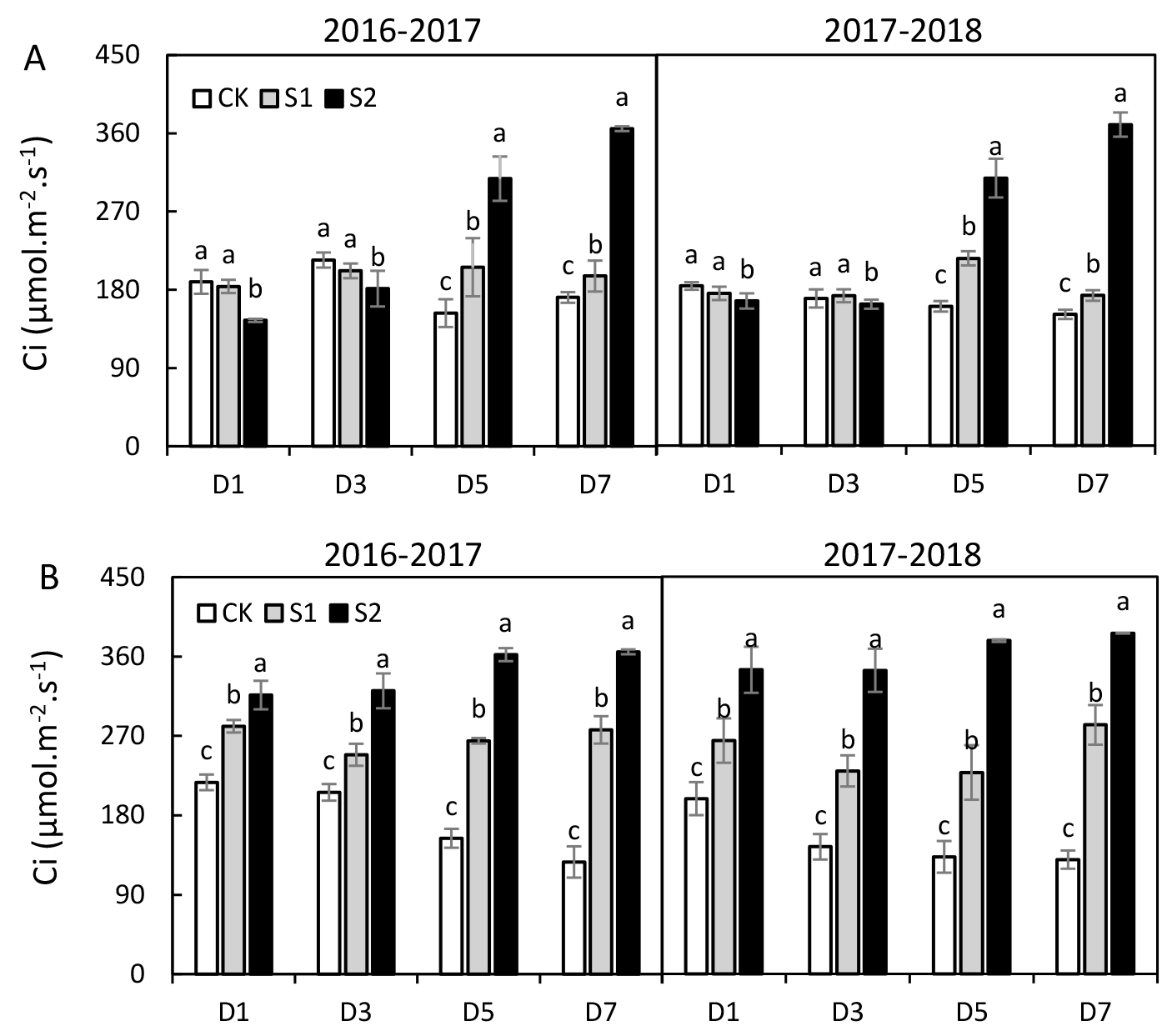

Supplement: Supplementary file 2 — Supplementary Information 2 [file 41598_2020_71268_MOESM2_ESM.tif]
